# Multi-omics insights into biomarkers of breast cancer associated diabetes: a computational approach

**DOI:** 10.3389/fmed.2025.1572500

**Published:** 2025-06-06

**Authors:** Tamizhini Loganathan, C. George Priya Doss

**Affiliations:** Laboratory of Integrative Genomics, Department of Integrative Biology, School of Bio Sciences and Technology, Vellore Institute of Technology (VIT), Vellore, Tamil Nadu, India

**Keywords:** breast cancer, diabetes, transcriptomics, exome analysis and TNF pathway, bioinformatics

## Abstract

**Introduction:**

Breast cancer (BC) and diabetes are multifaceted diseases with interconnected molecular mechanisms that are not yet fully elucidated. These diseases share common risk factors, biological pathways, and treatment outcomes.

**Methods:**

This study utilizes an integrative computational approach to investigate the interplay between BC and diabetes in African American (AA) and European American (EA) cohorts. It employs transcriptomic and exomic analyses to identify shared pathways and potential therapeutic targets.

**Results:**

The pooled cohort of differential expression analysis identified 2,815 genes differentially expressed in BC patients with diabetes compared to those without diabetes, including 1824 upregulated and 990 downregulated genes. We reanalyzed transcriptomic data by stratifying BC patients with and without diabetes into two cohorts, identifying 3,245 DEGs in AA and 3,208 DEGs in EA, with 786 genes commonly altered between both groups. Whole-exome sequencing (WES) of 23 BC patients with diabetes revealed 899 variants across 208 unique genes, predominantly missense mutations. Among these, nine key genes were prioritized, with *TNFRSF1B* (L264P) and *PDPN* (A105G) identified as the most deleterious variants. Functional enrichment analyses highlighted the significant involvement of pathways related to extracellular matrix organization, angiogenesis, immune regulation, and signaling processes critical to cancer progression and metabolic dysfunction. The TNF pathway emerged as a central link connecting chronic inflammation, insulin resistance, and tumor growth. TNF-mediated mechanisms, including NF-κB activation, oxidative stress, and epithelial-to-mesenchymal transition (EMT), were found to drive both diseases, promoting tumorigenesis, immune evasion, and metabolic dysregulation.

**Conclusion:**

This study provides critical molecular insights into the shared mechanisms of BC and diabetes, identifying the TNF pathway as a key therapeutic target to improve outcomes for patients with these interconnected conditions.

## 1 Introduction

Breast cancer (BC) is a multifaceted disease characterized by a wide range of genetic, molecular, and phenotypic variations (1). It remains one of the most prevalent malignancies among women worldwide, with significant heterogeneity in its clinical presentation, prognosis, and therapeutic response (2). Concurrently, diabetes, a chronic metabolic disorder characterized by hyperglycemia and insulin resistance, has been increasingly recognized as a comorbidity that influences cancer risk, progression, and treatment outcomes (3, 4). The intersection of BC and diabetes presents a unique and challenging clinical scenario that warrants a deeper understanding of the underlying molecular mechanisms and potential biomarkers (5). Diabetes has been implicated in altering the tumor microenvironment, enhancing chronic inflammation, promoting oxidative stress, and disrupting metabolic pathways, all of which can contribute to cancer initiation and progression (6, 7). The coexistence of diabetes with BC introduces additional layers of complexity, influencing tumor biology, therapeutic efficacy, and patient survival (3). Patients with diabetes are often associated with poor outcomes, including higher recurrence rates and reduced overall survival, potentially due to delayed diagnosis, altered pharmacokinetics of anticancer drugs, and the impact of hyperglycemia on cancer cell metabolism (8).

Advancements in high-throughput technologies, such as transcriptomics and exome sequencing, have significantly enhanced our ability to understand the molecular landscape of diseases (9). Exome sequencing facilitates the identification of somatic mutations, copy number variations, and other genomic alterations that drive cancer development (10). Conversely, transcriptomics provides insights into gene expression patterns, revealing dysregulated pathways and potential therapeutic targets (11, 12). Integrating transcriptomics and exome data has proven to be a powerful approach to uncover genetic and transcriptomic alterations, providing a more comprehensive understanding of the molecular mechanisms driving conditions such as cancer and other diseases. This integration has the potential to identify novel biomarkers and therapeutic targets. Biomarkers are invaluable for stratifying patients, predicting therapeutic responses, and monitoring disease progression (13–15). Few studies have explored the diabetes-associated gene expression profiles in BC, revealing the unique signatures that could be targeted therapeutically or used as diagnostic tools (16–19). Understanding the molecular interplay between BC and diabetes can pave the way for personalized medicine approaches, ensuring more effective and tailored treatments.

**Figure 1:**
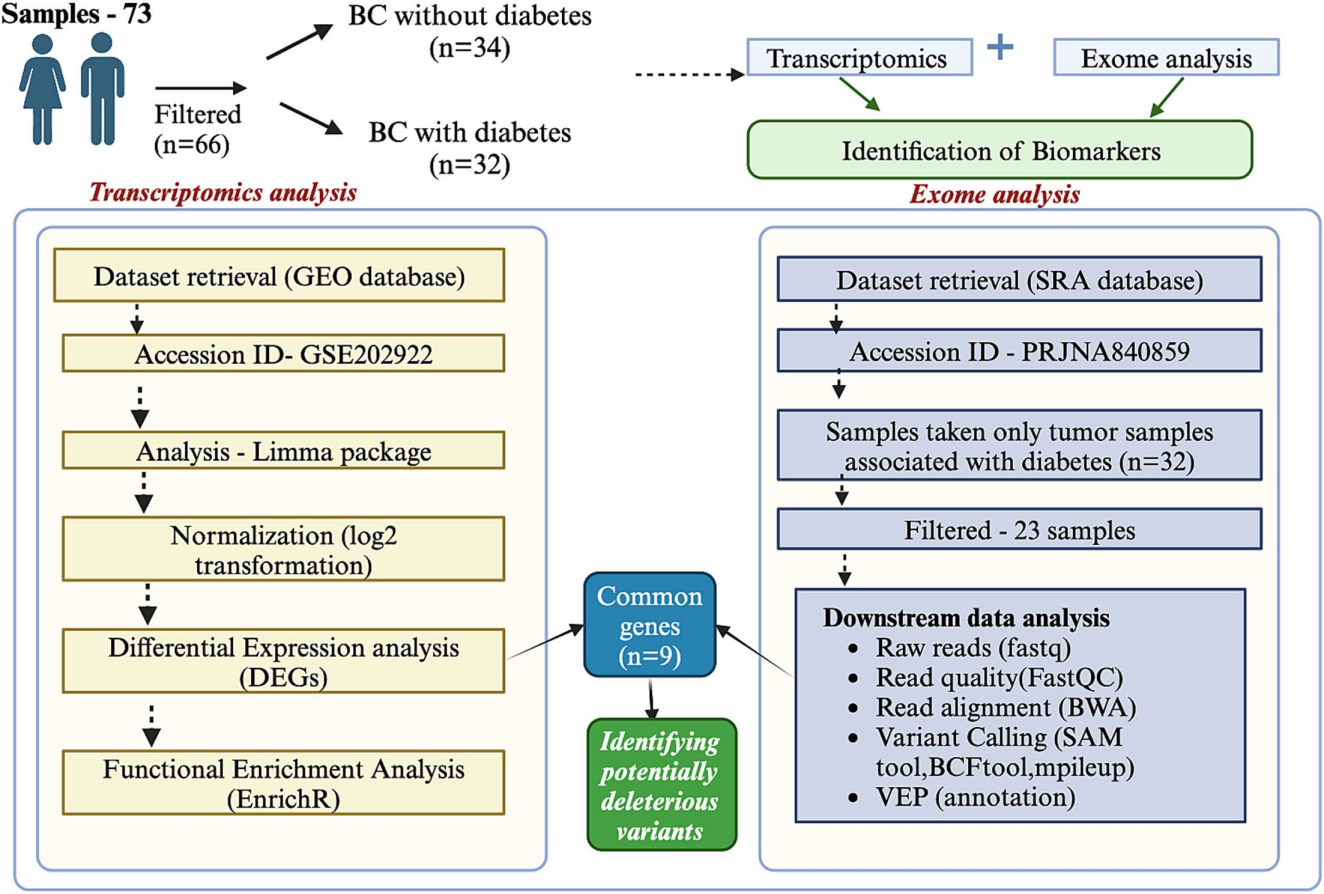
Integration of transcriptomics and exome data analysis. The figure illustrates the workflow and outcomes of integrating transcriptomics and exome data analysis. Transcriptomics data provides insights into differential gene expression across conditions, while exome data reveals coding region mutations. The integration identifies overlapping features, including genes with significant expression changes and mutations. This combined approach highlights key biomarkers, potential driver genes, and pathways associated with the biological process of interest.

In this study, we aim to explore the biomarker landscape in BC with diabetes (African American (AA) and European American (EA) cohorts) through a comprehensive analysis of transcriptomics and exome data. By examining the transcriptomic and genomic profiles specific to this cohort, we seek to identify key molecular players and pathways that underlie the interaction between these two conditions. Our findings could provide insights into the mechanistic basis of BC in diabetic patients, highlight potential therapeutic vulnerabilities, and contribute to the development of precision oncology strategies. The detailed workflow is illustrated in Figure 1.

## 2 Materials and methods

### 2.1 Transcriptomics data analysis

All data used in this study were obtained from the NCBI database. We acquired the gene expression profiling dataset produced through high-throughput sequencing (GSE202922) (16) using Illumina HiSeq 3,000 from the publicly available GEO database (20). The dataset has a total of 73 samples, and a further 66 samples have raw counts. A total of 66 samples were included in this study, comprising 32 diabetic and 34 non-diabetic cases. The detailed metadata information, along with transcriptomics data of the 66 samples, were described in Supplementary Table 1 and Supplementary Figure 1A. We also conducted race-specific transcriptomic analyses using datasets from African American (AA) and European American (EA) cohorts. The metadata for these cohorts is provided in Supplementary Table 2. GEO2R is a web-based analysis tool that enables user to compare multiple sample groups within a GEO Series to find deregulated genes under certain experimental conditions (21). Moreover, differentially expressed genes (DEGs) were detected using the limma R package (22), applying a threshold of |log2FoldChange| > = 0.5, adj *p* < =0.05, and *p* < = 0.05. All statistical analyses and data visualization were carried out using R/Bioconductor packages. Statistical plots such as boxplot and UMAP plot were performed and analyzed.

### 2.2 Exome data analysis

The study also utilized Whole Exome Sequencing (WES) data with ID: PRJNA840859 comprising 32 individuals with BC-associated diabetes (16). Supplementary Figure 1B provides detailed information on the selected exome data. After verifying the availability of exome data, 23 sample reads were retrieved and analyzed. These samples were subjected to exome sequence analysis. Sequencing was performed on Illumina NovaSeq 6,000 systems, generating paired-end reads. A shell script was employed to download the sequencing reads from the ENA database (23). The exome sequencing pipeline involves a comprehensive workflow for processing, analyzing, and interpreting genetic data to ensure high accuracy and reliability in identifying variants. The process begins with quality control using FastQC (24), which evaluates critical metrics such as read quality scores, GC content, and adapter contamination. This step helps to identify potential issues in the raw FASTQ files, ensuring only high-quality reads proceed to the next stage. Tools like Trimmomatic remove low-quality bases and adapter sequences in the read preprocessing step. Reads with quality scores below a threshold (commonly Q30) are trimmed or discarded, producing a clean dataset suitable for downstream analysis. Next, the high-quality reads are aligned to the human reference genome GRCh38 (25) using the BWA-MEM algorithm (26), a widely used tool for efficient and accurate alignment of short-read sequences. This step generates SAM files containing mapped reads and their corresponding positions on the genome. These SAM files are converted into BAM format using SAMtools, sorted by coordinate order, and indexed to enable efficient querying and visualization in downstream applications. The variant calling step identifies genetic variants such as SNPs and indels (27). BCFtools generate a pileup of aligned reads, and variants are called highly confidently (28). The resulting data is output in the Variant Call Format (VCF), which contains detailed information about each identified variant.

Once variants are called, they undergo filtering and annotation. Each sample VCF was merged using the “VCFmerge tool” and the Galaxy tool. The Ensembl Variant Effect Predictor (VEP) was used to annotate the functional consequences of genes (29). Filtering ensures that only high-confidence variants are retained by removing low-quality or potentially false-positive calls. Tools like the BCFtools filter allow for applying stringent criteria, such as minimum quality scores or read depth thresholds. Annotating the filtered variants with databases such as dbSNP and ClinVar provides functional insights, including potential pathogenicity, population frequency, and relevance to known diseases. The missense variants were retrieved and further used for functional analysis.

### 2.3 Functional enrichment analysis

Functional analysis of the differentially expressed genes (DEGs) identified from the transcriptomic analysis was conducted using EnrichR (30). Additionally, common genes identified from both transcriptomic and exome analyses were analyzed. Functional enrichment analysis included Gene Ontology categories: Biological Process (GO-BP), Cellular Component (GO-CC), and Molecular Function (GO-MF), as well as pathway analyses using KEGG and Reactome. Protein-coding genes with a *p*-value < 0.05 were used as the background gene set.

### 2.4 Identification of potentially deleterious variants

Genes featuring missense variants from a curated in-house list of cancer-associated genes were subsequently examined for functional effects using the PredictSNP web tool (31). This examination utilized six well-known predictive tools, MAPP, PhD-SNP, PolyPhen-1, PolyPhen-2, SIFT, and SNAP, to detect potentially harmful variants (missense).

MAPP demonstrated that the likelihood of disease or cancer risk is closely linked to breaches of physicochemical limitations due to amino acid variations (32). PhD-SNP, based on support vector machines (SVMs), was used to determine whether a given point mutation was a neutral polymorphism or associated with genetic disorders (33). PolyPhen-1 analyzed the impact of missense variants on protein structure and function (34). In contrast, PolyPhen-2 incorporated both sequence- and structure-based features, utilizing a Naïve Bayesian classifier to predict the consequences of amino acid substitutions. Variants identified as “probably damaging” or “possibly damaging” (scores ≥0.5) were categorized as harmful, whereas “benign” variants (scores <0.5) were regarded as acceptable. Scores nearer to 1.0 were more prone to be damaging (35).

SIFT predicted the potential harm of variants using a normalized probability score, where scores <0.05 were deemed harmful and scores ≥0.05 were considered neutral. The SIFT score assessed the effect of amino acid substitutions on protein function (36). SNAP was used to evaluate the functional impact of missense variants (37). Protein stability alterations due to single-point variants were forecasted using I-Mutant 2.0, which categorized variants into two groups: reduced stability (<0 kcal/mol -decrease) and enhanced stability (>0 kcal/mol – increase) (38).

The evolutionary conservation of amino acid positions for the most deleterious variants were assessed using the ConSurf online tool. Conservation scores ranges from 1 (most variable positions) to 9 (most conserved positions), providing insights into the variants’ functional significance (39).

## 3 Results

### 3.1 Transcriptomics and functional analysis of pooled cohort

**Figure 2:**
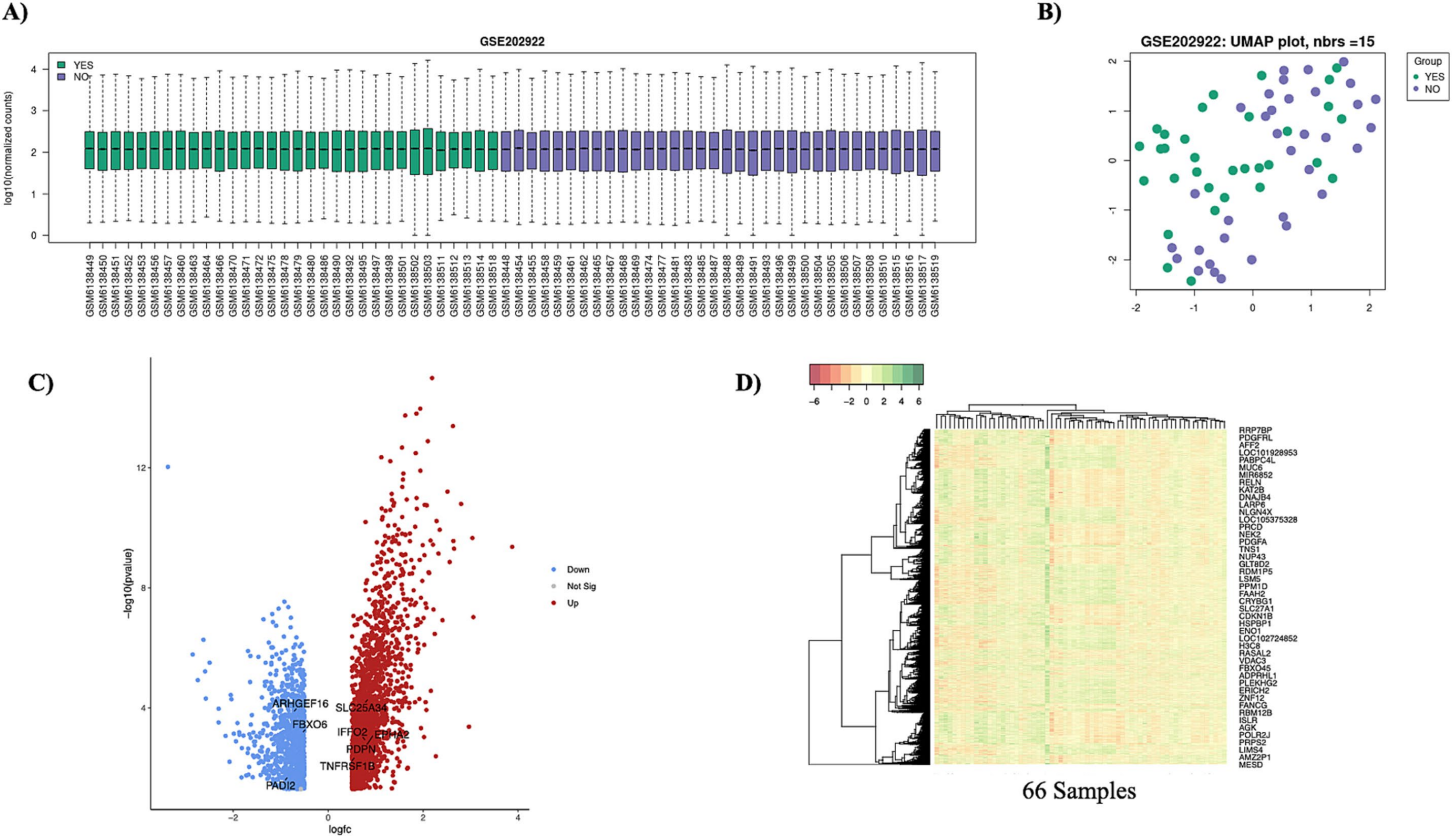
Statistical plots of transcriptomics data. **(A)** The boxplot represents the distribution of normalized transcriptomics data across all samples. Each box corresponds to an individual sample, with the central line representing the median expression level. **(B)** The UMAP plot illustrates the clustering of transcriptomics data, with each point representing an individual sample. Samples are color-coded based on their respective groups (BC with diabetes vs. BC without diabetes). This visualization highlights the underlying structure and relationships in the dataset, revealing group-specific patterns. The “YES” label represents the BC with diabetes, and the “NO” label represents the BC without diabetes. The color-coded representation of the “YES” label is green, and the “NO” label is purple. **(C)** The volcano plot shows the relationship between statistical significance for all genes. Significant upregulated and downregulated genes are highlighted in distinct colors with respective thresholds. This visualization identifies key differentially expressed genes. The red denotes the upregulated genes, and the blue indicates the down-regulated genes. **(D)** The heatmap visualizes the expression levels of selected genes across 66 samples. Rows represent genes, and columns represent samples.

To identify differentially expressed genes (DEGs) between BC patients with and without diabetes, we utilized normalized expression data from the GEO database. The GEO2R tool, based on the limma package, was employed for the analysis. 2,814 DEGs were analyzed across 66 samples, including BC without and BC with diabetes samples. A boxplot is a graphical representation of the distribution of a dataset that shows its central tendency and variability. It provides a concise summary of the data’s statistical properties across samples. The boxplot of each group comparison is mentioned in the Figure 2A. A UMAP plot is a dimensionality reduction technique that is particularly useful for visualizing high-dimensional data, such as gene expression values plotted in Figure 2B. The comparison revealed 2,814 DEGs comprising 1824 upregulated and 990 downregulated genes (*p*-value <= 0.05, adj *p*-value <= 0.05, |log2 fold change| > =0.5). The DEGs were visualized using a volcano plot (Figure 2C), highlighting significant genes with biological relevance. The detailed results of DEGs are mentioned in Supplementary Table 3. The heatmap illustrates the expression levels of selected genes across 66 samples, with rows representing genes and columns representing samples, as mentioned in Figure 2D.

**Figure 3:**
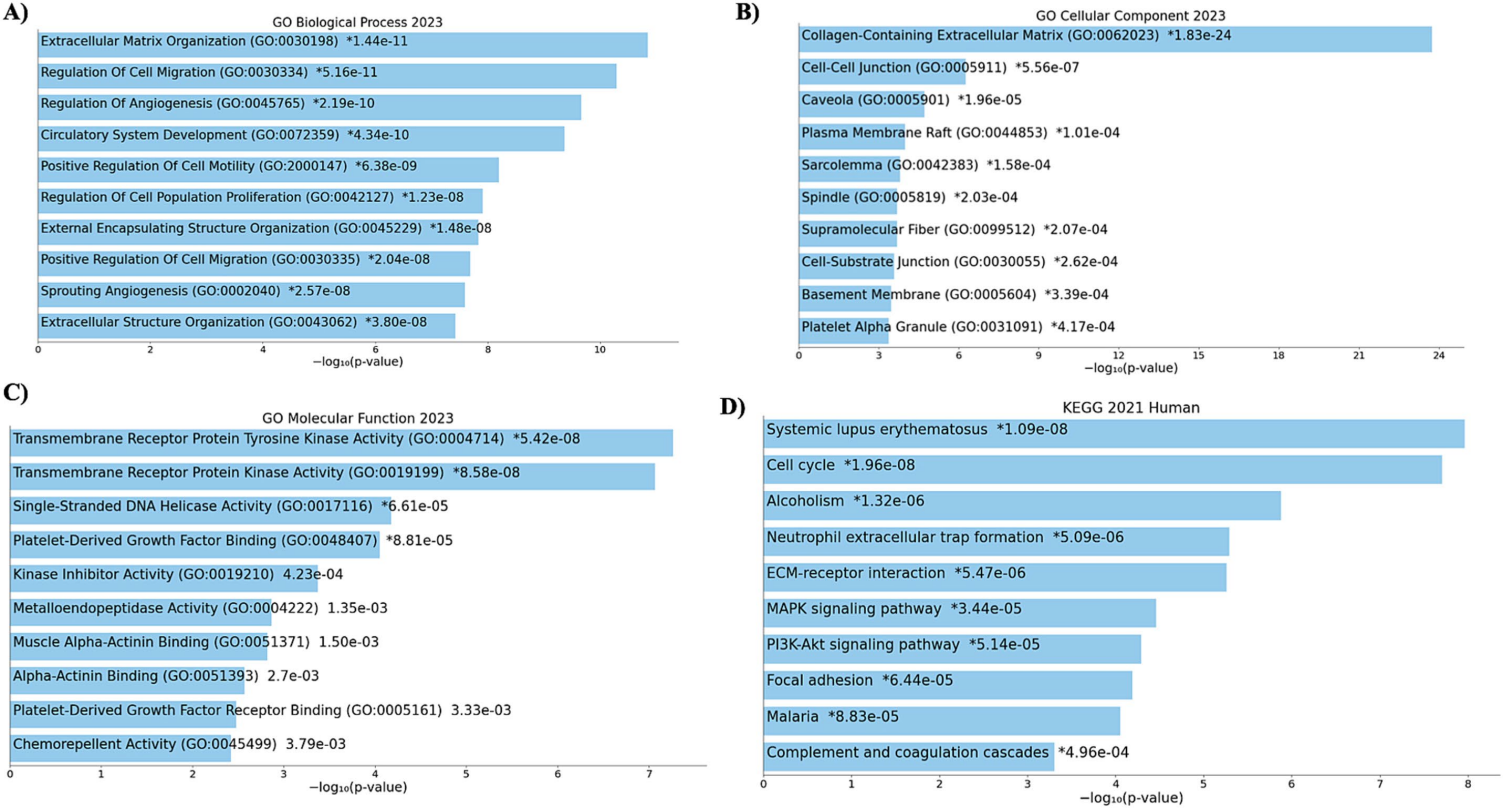
Functional enrichment analysis (transcriptomics). The figure showcases the functional enrichment analysis of differentially expressed genes (DEGs) derived from transcriptomics data. **(A)** GO-BP, **(B)** GO-CC, **(C)** GO-MF, and **(D)** KEGG pathways. Bar sizes indicate statistical significance (adjusted *p*-values) and gene ratios, providing insights into DEGs’ molecular and functional context.

The functional enrichment analysis of 2,814 DEGs was performed using EnrichR. The background genes are protein-coding genes with *p*-value<=0.05. The functional terms are GO (Gene ontology) terms and KEGG pathways. Significant enrichment is seen in processes such as extracellular matrix organization, regulation of cell migration, angiogenesis, and circulatory system development. These are key processes in tissue remodeling, cancer metastasis, and vascular development. Highlighted components include collagen-containing extracellular matrix, cell junctions, plasma membrane raft, and sarcolemma. These components are critical for cellular integrity, signaling, and intercellular communication. Functions such as tyrosine kinase activity, platelet-derived growth factor binding, and kinase inhibitor activity dominate. These molecular functions are often associated with signaling pathways and therapeutic targets in cancer and other diseases. Enriched pathways include systemic lupus erythematosus, cell cycle regulation, ECM-receptor interaction, and PI3K-Akt signaling. These pathways are relevant to immune disorders, cancer progression, and extracellular matrix interactions. The results suggest an association with processes and pathways related to cancer progression, immune regulation, and extracellular matrix dynamics. The length of the bar indicates the top function in the barplot. The detailed functional results are mentioned in Figure 3.

### 3.2 Transcriptomics and functional analysis of AA and EA cohorts

To identify DEGs between BC patients with and without diabetes, we analyzed normalized expression data from the GEO database separately for African American (AA) and European American (EA) cohorts. The analysis was performed using the GEO2R tool, which is based on the limma package. In the African American (AA) cohort, a total of 3,245 differentially expressed genes (DEGs) were identified from 57 samples, including 1,922 upregulated and 1,323 downregulated genes, based on thresholds of *p*-value ≤ 0.05, adjusted *p*-value ≤ 0.05, and |log₂ fold change| ≥ 0.5. Similarly, in the European American (EA) cohort, 3,208 DEGs were detected across 17 samples, with 1,640 genes upregulated and 1,568 downregulated using the same statistical criteria. Notably, 786 DEGs were found to be shared between the AA and EA cohorts. The statistical plots of boxplot and UMAP were performed and mentioned in Supplementary Figure 2. The detailed information of DEGs of both the cohorts were mentioned in the Supplementary Tables 4, 5.

The functional enrichment analysis of each cohort was performed. The functional enrichment analysis of 3,245 (AA cohort) and 3,208 (EA cohort) DEGs was performed using EnrichR. The background genes are protein-coding genes with *p*-value<=0.05. Some of the KEGG’s significant functions are cell cycle, ECM receptor interactions, PI3K-Akt signaling, and AGE-RAGE signaling pathway in diabetic complications, and these functions were specific to the AA cohort. Some of the important functions in the EA cohort are Oxidative phosphorylation and Diabetic cardiomyopathy. The detailed information on these enrichment analyses is mentioned in Supplementary Figures 3, 4.

The Venn diagram illustrates the overlap in transcriptomic data between the African American (AA) and European American (EA) cohorts, revealing 786 genes common to both groups (Supplementary Figure 5A). Further functional analysis of GO-BP,GO-CC,GO-MF and KEGG pathways were performed on common genes. Some of the important functions are Notch signaling pathway and Hippo signaling pathway. Both these functions were related to BC and diabetes. The detailed functional enrichment analysis were mentioned in the Supplementary Figures 5B–E.

### 3.3 Exome data analysis

We retrieved WES datasets for BC with diabetes from the NCBI SRA database. The tumor data of BC with diabetes (*n* = 23) were only taken for further analysis. Each sample was processed using a computational pipeline tailored to laboratory protocols. Sequence quality was assessed using the FastQC tool. High-quality data for analysis was ensured by trimming low-quality reads, removing adapters, and further validating the sequences’ base quality. Following the evaluation of read quality, the final reads were mapped to the human reference genome GRCh38.p13 (hg38) utilizing the BWA aligner with default settings. Every dataset attained a total alignment rate surpassing 85%. SAM tools were employed to process and enhance the sequenced files further in the “SAM” format. The SAM files were first transformed into BAM format by utilizing the “samtools view” command. This transformation enabled later processes, including file sorting, indexing, and arranging mapped reads for further analysis. Prior to indexing, samtools organized the aligned reads and clustered them according to particular genomic areas. In conclusion, base calls from the mapped reads aligned to the reference sequence were compiled using the “samtools mpileup” command.

**Figure 4:**
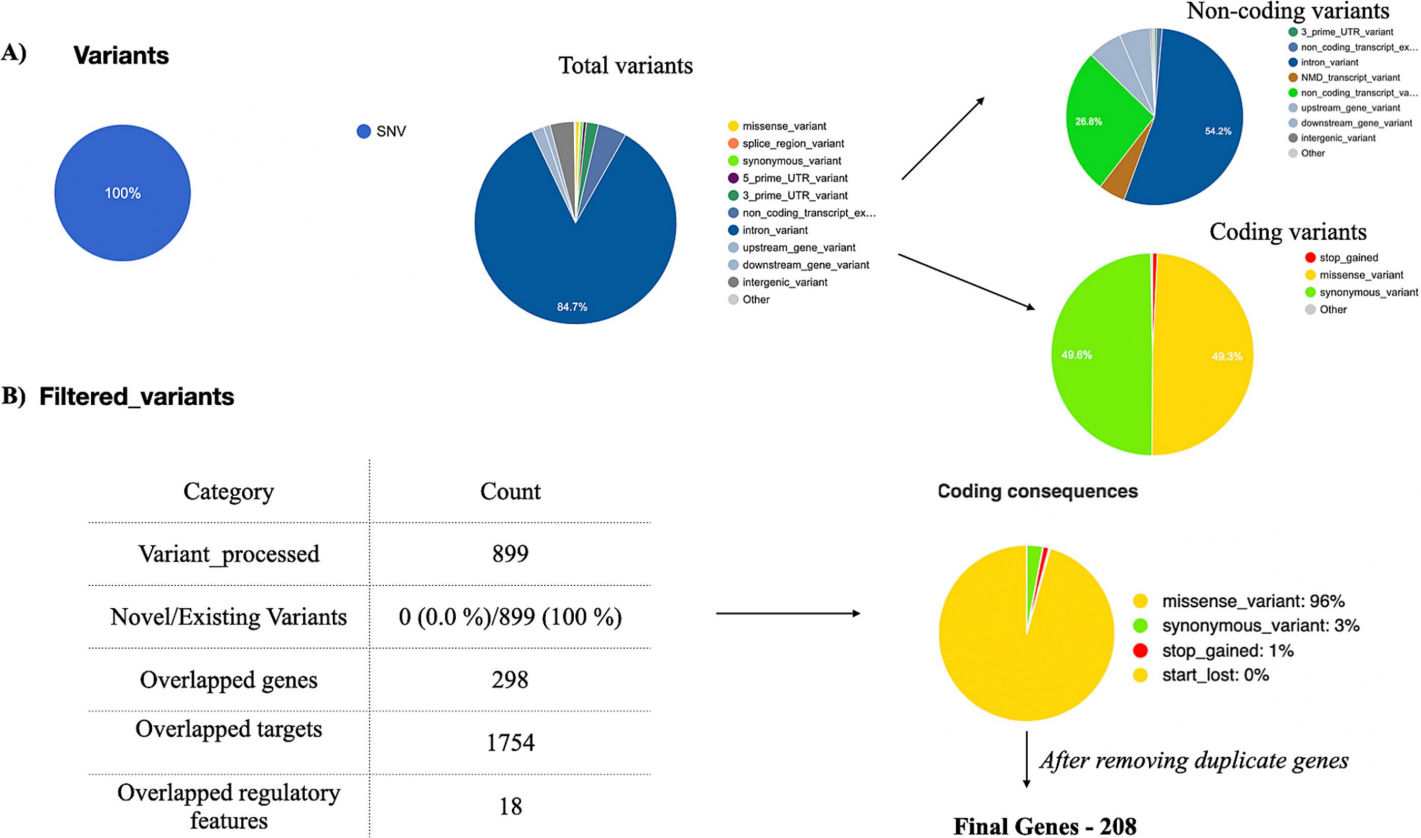
Exome data analysis. Exome data analysis was conducted on tumor samples (*n* = 23) from breast cancer patients with diabetes. Variants were annotated using the VEP tool. **(A)** A pie chart was generated to classify coding and non-coding variants. **(B)** Among the filtered coding variants, 96% were identified as missense variants. A total of 208 missense variants, derived from all 23 samples, were selected for further analysis.

**Figure 5:**
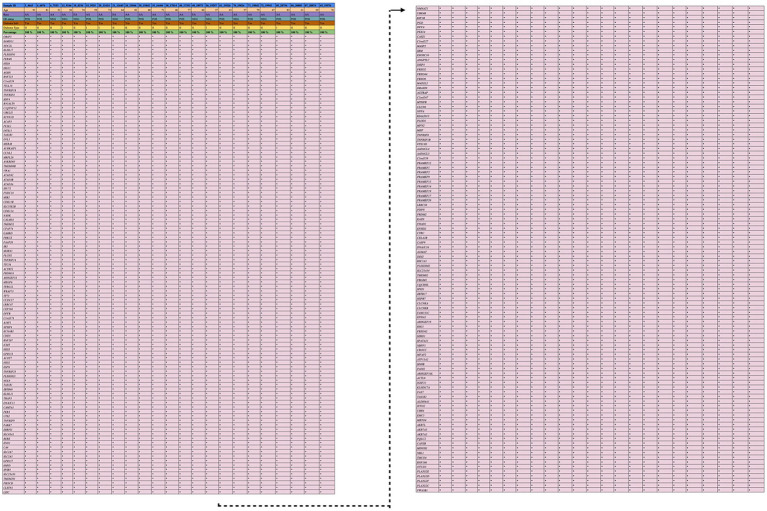
Distribution of variants. This figure illustrates the distribution of genetic variants observed in 23 samples, stratified by metadata variables such as age, race, ER status, diabetes type, and diabetes info. Each sample is represented as a distinct bar or point, categorized by metadata groups.

After processing the output from “mpileup” with BCFtools, SNPs in relation to the reference genome were identified and interpreted as variations. The VCF and its binary counterpart, BCF, were used in the analysis to handle the data. The resulting output for each dataset was provided in a VCF format, containing detailed information about variant positions, types, and quality. Each VCF file was annotated using the Ensembl VEP database (release 113) which provided a thorough analysis of the variants detected in each sample. All identified variants are single-nucleotide (SNVs), accounting for 100% of the dataset. There are two types of variants: non-coding variants and coding variants. The non-coding variants constitute 56.9% of the total, including regions like upstream, downstream, and intronic variants. The coding variants represent 43.1%, further categorized into missense variants 96%, synonymous variants 3%, stop-gained 1%, and no start-lost variants. The results are depicted in Figure 4A. Among the 3,238 observed missense variants in the VCF file, filtered 899 missense variants were chosen for further analysis (no novel variants were detected) (Figure 4B). After removing duplicate genes in the 298 overlapped genes, 208 unique genes were finalized for further analysis. The detailed results of missense variants are mentioned in Supplementary Table 6. The distribution of 208 genes with respective metadata is depicted in Figure 5.

### 3.4 Identification of shared genes and their respective functional analysis

**Figure 6:**
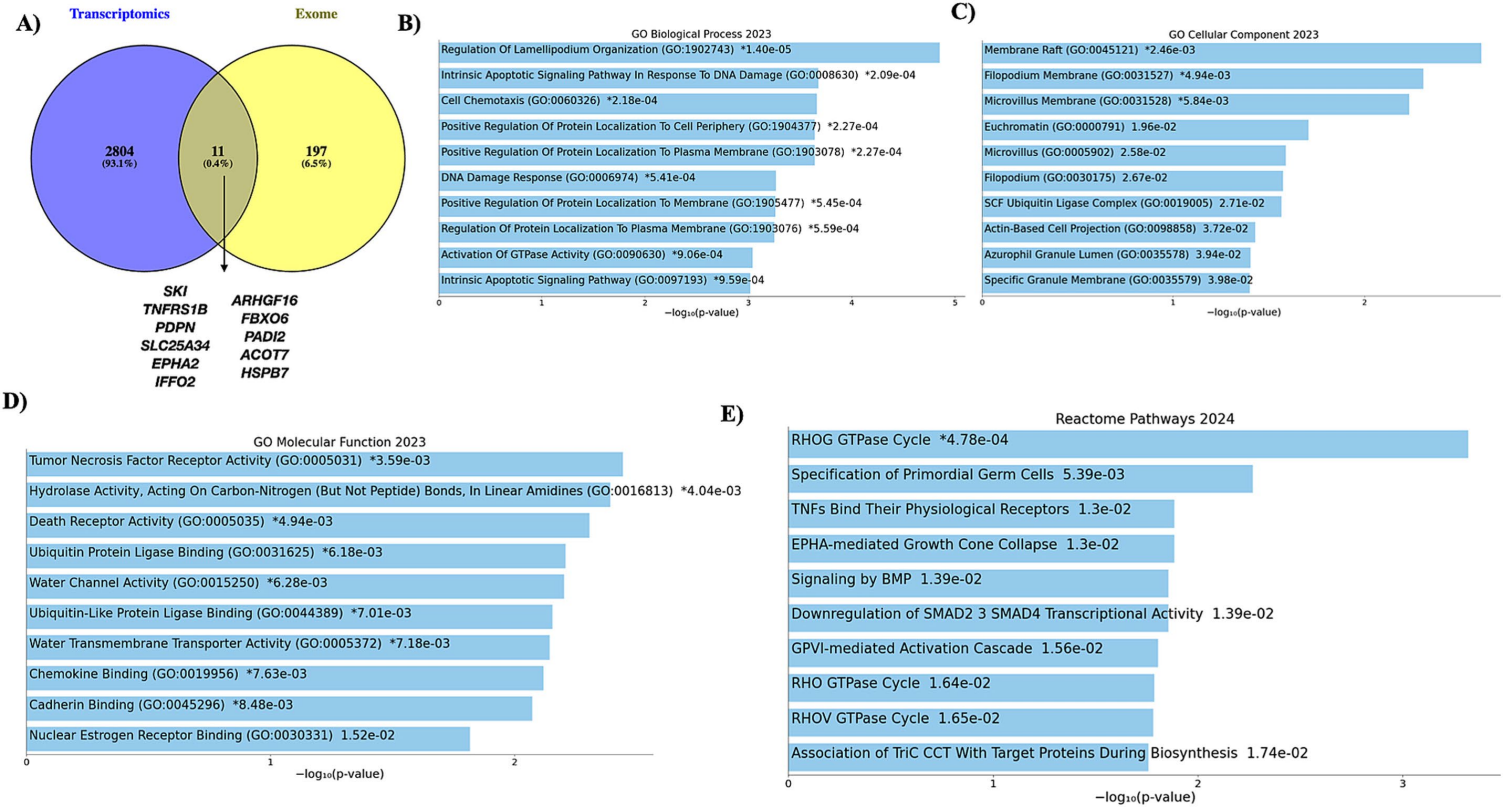
Overlapping genes and functional analysis. This figure shows the overlapping genes identified through integrative analysis of exome sequencing and transcriptomics data. The shared genes represent a subset with potential biological functions. **(A)** Eleven genes overlapped the exome and transcriptomics data. The functional analysis of common genes was performed. **(B)** GO-BP, **(C)** GO-CC, and **(D)** GO-MF. **(E)** Reactome pathways.

The Venn diagram illustrates the overlap between transcriptomic and exomic data. A total of 2,804 unique genes were identified only in transcriptomic analysis. One hundred ninety-seven unique genes are found exclusively in the exomic data. Eleven genes are shared between the datasets, representing key potentially important genes across transcriptional and mutational levels. The two genes with no variations were excluded from the analysis. The nine genes comprises six upregulated genes (*SKI, TNFRSF1B, PDPN, SLC25A34, EPHA2,* and *IFFO2*) and three down-regulated genes (*ARHGEF16, FBXO6,* and *PADI2*). The results of overlap genes are mentioned in Figure 6A. We compared the selected genes across two different cohorts AA and EA populations. Four genes such as *SKI, TNFRSF1B, SLC25A34,* and *EPHA2,* were present in both cohorts.

The functional analysis of nine genes was performed and analyzed, including the functional categories such as GO-BP, GO-CC, GO-MF, and the Reactome pathway. The GO-BP enriched terms include protein localization processes and synaptic pathways, reflecting cellular organization and signaling roles. The GO-CC, the enriched components, such as synaptic and endosomal compartments, highlight cellular compartmentalization for signaling and transport. The GO-MF of enriched functions includes ubiquitin-protein ligase binding, TNF activity, chemokine activity, and cadherin binding, which are crucial for protein regulation and cellular interactions. The Reactome pathways enriched are RHOG GTPase cycle, TNFs Bind their physiological receptors. The other pathways include EPHA-mediated growth cone collapse and signaling pathways. The functional results are mentioned in the Figures 6B–E. These variations were taken for further analysis.

### 3.5 Identification of potential variants

The analysis involved nine genes, focusing on identifying the most deleterious variants using a comprehensive suite of online prediction tools. These tools included PredictSNP, MAPP, PhD-SNP, PolyPhen-1, PolyPhen-2, SIFT, SNAP, and PANTHER, each offering distinct methodologies for assessing variant pathogenicity. The prediction results provided detailed insights into the potential impact of these variants on protein function and structure. A summary of the findings, highlighting the pathogenicity scores from each tool for the identified variants, is presented in Table 1. This table serves as a consolidated resource, showcasing the comparative outcomes from all tools, thus facilitating an in-depth evaluation of the most deleterious genetic changes. Among these nine genes, the TNFRSF1B (L264P) and PDPN (A105G) were the top 2 variants predicted by the above tools.

**Table 1:** Prediction of deleterious variants of common genes by different tools.

Protein	Uniport ID	Amino_acid_change	Existing_variation	PredictSNP prediction	MAPP prediction	PhD-SNP prediction	PolyPhen-1 prediction	PolyPhen-2 prediction	SIFT prediction	SNAP prediction	PANTHER prediction
SKI	P12755	A62G	rs28384811	Neutral	Neutral	Neutral	Neutral	Deleterious	Deleterious	Neutral	Unknown
SKI	P12755	E491D	rs1266460001	Neutral	Neutral	Neutral	Neutral	Deleterious	Neutral	Neutral	Neutral
TNFRSF1B	P20333	M196R	rs1061622	Neutral	Deleterious	Neutral	Neutral	Neutral	Neutral	Neutral	Neutral
TNFRSF1B	P20333	E232K	rs5746026	Neutral	Deleterious	Neutral	Neutral	Neutral	Neutral	Neutral	Neutral
TNFRSF1B*	P20333	L264P	rs2229700	Deleterious	Deleterious	Deleterious	Deleterious	Neutral	Deleterious	Deleterious	Deleterious
PDPN	Q86YL7	M43V	rs141726617	Neutral	Neutral	Neutral	Neutral	Neutral	Neutral	Neutral	Neutral
PDPN*	Q86YL7	A105G	rs2486188	Neutral	Deleterious	Neutral	Deleterious	Deleterious	Neutral	Neutral	Neutral
PDPN	Q86YL7	A147G	rs2486188	Neutral	Neutral	Neutral	Neutral	Neutral	Neutral	Neutral	Neutral
SLC25A34	Q6PIV7	I215M	rs62621224	Neutral	Deleterious	Deleterious	Neutral	Neutral	Neutral	Neutral	Neutral
EPHA2	P29317	V747I	rs145592908	Neutral	Neutral	Deleterious	Neutral	Deleterious	Neutral	Neutral	Unknown
EPHA2	P29317	M631T	rs34021505	Neutral	Neutral	Neutral	Deleterious	Neutral	Neutral	Neutral	Neutral
EPHA2	P29317	V541M	rs61731097	Neutral	Neutral	Neutral	Neutral	Neutral	Deleterious	Neutral	Neutral
EPHA2	P29317	G391R	rs34192549	Neutral	Na	Neutral	Deleterious	Neutral	Deleterious	Neutral	Neutral
EPHA2	P29317	D232G	rs114498261	Neutral	Neutral	Neutral	Neutral	Neutral	Neutral	Neutral	Neutral
IFFO2	Q5TF58	V352I	rs6675316	Neutral	Neutral	Neutral	Neutral	Deleterious	Neutral	Neutral	Neutral
ARHGEF16	Q5VV41	V137M	rs3806164	Neutral	Neutral	Neutral	Neutral	Neutral	Neutral	Neutral	Deleterious
ARHGEF16	Q5VV41	H370Y	rs2185639	Neutral	Neutral	Deleterious	Neutral	Neutral	Neutral	Neutral	Deleterious
FBXO6	Q9NRD1	R60Q	rs3125818	Neutral	Neutral	Neutral	Neutral	Deleterious	Neutral	Neutral	Neutral
FBXO6	Q9NRD1	V72M	rs766167101	Neutral	Neutral	Neutral	Neutral	Neutral	Neutral	Neutral	Unknown
FBXO6	Q9NRD1	V290I	rs140436527	Neutral	Na	Neutral	Neutral	Neutral	Neutral	Neutral	Unknown
PADI2	Q9Y2J8	Y275H	NA	Neutral	Neutral	Neutral	Deleterious	Deleterious	Neutral	Neutral	Neutral
PADI2	Q9Y2J8	D259N	rs150731573	Neutral	Neutral	Deleterious	Neutral	Neutral	Deleterious	Neutral	Neutral

* Represents the most deleterious variants from all the tools.

**Figure 7:**
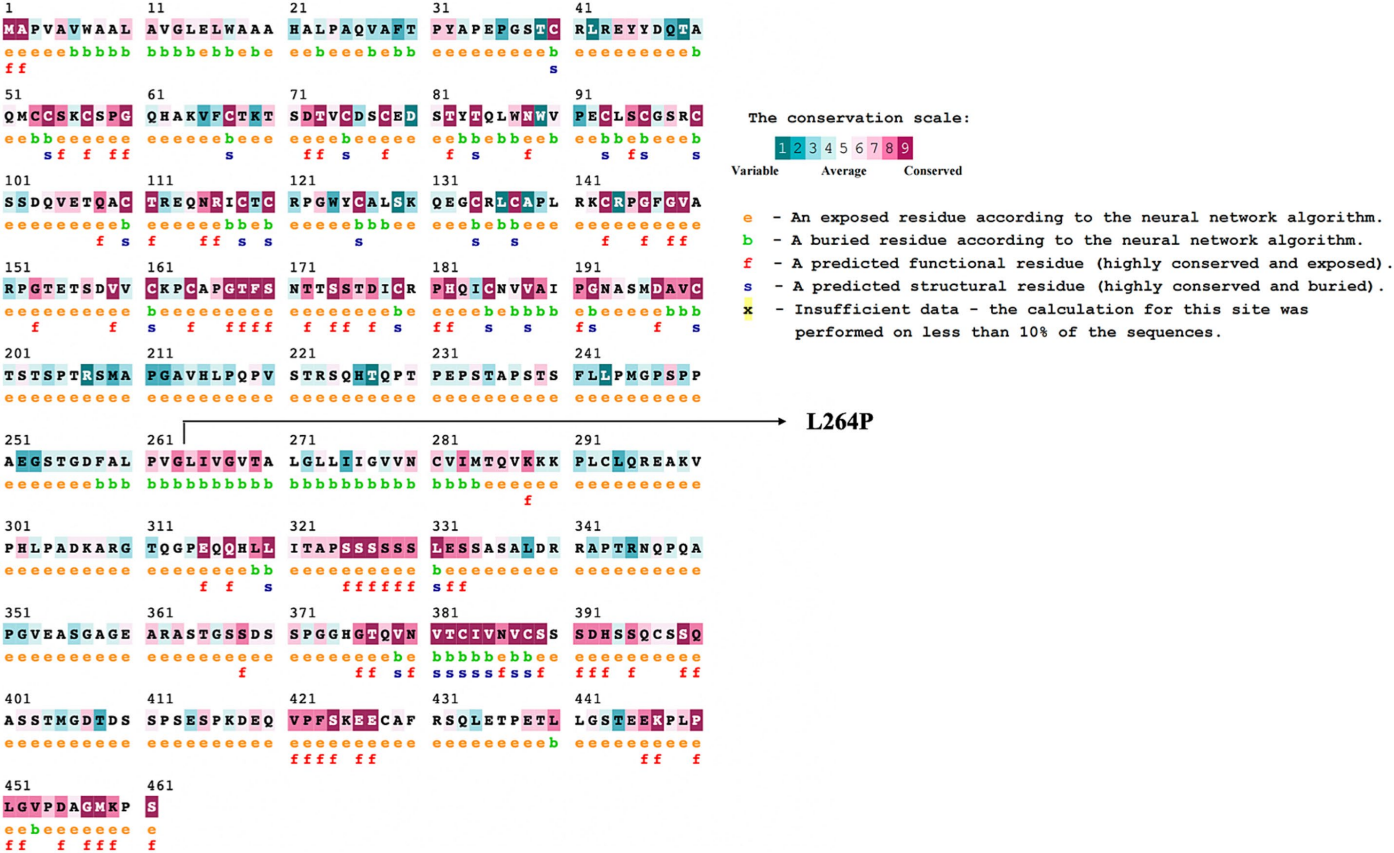
ConSurf analysis of potential deleterious variant (TNFRSF1B-L264P). This figure presents the results of a ConSurf analysis, highlighting the evolutionary conservation of amino acid residues in the TNFRSF1B (L264P) protein. Residues are color-coded based on their conservation scores, ranging from highly conserved (dark shades) to variable (light shades).

Variants classified as neutral were excluded from stability analysis using I-Mutant 2.0. analysis. Among these nine gene variants, the results revealed distinct patterns of stability changes. Three gene variants exhibited an increase in protein stability upon mutation. This indicates that these mutations potentially enhance the structural integrity or thermodynamic stability of the proteins, which could impact their functional roles positively or negatively, depending on the biological context. The remaining six gene variants showed a decrease in protein stability upon mutation. A reduction in stability suggests that these mutations may disrupt the protein’s structural conformation, potentially leading to misfolding, aggregation, or loss of function (38). Such destabilizing mutations could contribute to disease pathogenesis or altered protein activity. The results are mentioned in Table 2. Among these mutations, the TNFRSF1B variant (L264P) is the most deleterious variant confirmed by all computational tools. ConSurf is a tool that analyses the evolutionary conservation of amino acid positions in protein sequences. The variant (TNFRSF1B-L264P) is categorized as a highly conserved position with a significant score (score range of 9), it may suggest a deleterious impact. The ConSurf results are mentioned in Figure 7.

**Table 2:** Prediction of protein stability using I-Mutant 2.0.

Protein	Uniport ID	Amino_acid_change	Existing_variation	Stability	RI	DDG_value (kcal/mol)
SKI	P12755	A62G	rs28384811	Decrease	5	−0.28
SKI	P12755	E491D	rs1266460001	Increase	6	0.04
TNFRSF1B	P20333	M196R	rs1061622	Decrease	7	−1.07
TNFRSF1B	P20333	L264P	rs2229700	Decrease	4	−2.29
PDPN	Q86YL7	A105G	rs2486188	Decrease	7	−1.68
SLC25A34	Q6PIV7	I215M	rs62621224	Decrease	7	−1.91
EPHA2	P29317	V747I	rs145592908	Decrease	7	−0.57
EPHA2	P29317	M631T	rs34021505	Increase	1	0.1
EPHA2	P29317	D232G	rs114498261	Decrease	3	−0.9
IFFO2	Q5TF58	V352I	rs6675316	Decrease	8	−1.04
ARHGEF16	Q5VV41	V137M	rs3806164	Decrease	7	−2.43
ARHGEF16	Q5VV41	H370Y	rs2185639	Increase	4	2.02
FBXO6	Q9NRD1	R60Q	rs3125818	Decrease	8	−0.94
PADI2	Q9Y2J8	Y275H	NA	Decrease	6	−0.62
PADI2	Q9Y2J8	D259N	rs150731573	Decrease	0	−0.56

## 4 Discussion

Integrating transcriptomics and exomic analyses combines the strengths of both methods to achieve a comprehensive understanding of genomic and transcriptomic changes in biological systems (40, 41). This integration represents a powerful approach to elucidating the molecular mechanisms underlying complex diseases, facilitating the identification of robust biomarkers and therapeutic targets (42). Several studies have successfully integrated transcriptomics and exomic data to provide deeper insights into biological mechanisms, disease pathogenesis, and therapeutic strategies (43–47). The etiology of BC associated with diabetes remains poorly understood. We aimed to identify differentially expressed genes (DEGs) between BC patients without diabetes and those with diabetes. Our analysis included 66 samples, comparing BC without diabetes to BC with diabetes, and identified 2,815 DEGs, comprising 1,824 upregulated and 990 downregulated genes with statistical significance. This integrative analysis provides insights into the gene expression changes associated with diabetes in BC patients, with visualizations effectively summarizing statistical properties, significant DEGs, and their expression patterns.

Functional enrichment analysis of the 2,814 DEGs was performed using EnrichR, with protein-coding genes as the background. The study focused on GO terms and KEGG pathways, revealing significant enrichment in processes and pathways related to cancer progression, immune regulation, and extracellular matrix (ECM) dynamics. Notable enrichment was observed in processes such as extracellular matrix organization, regulation of cell migration, angiogenesis, and circulatory system development. These processes are crucial for tissue remodeling, cancer metastasis, and vascular development. Key components highlighted include collagen-containing extracellular matrix, cell junctions, plasma membrane rafts, and sarcolemma, which are essential for cellular integrity, signaling, and intercellular communication. Functions like tyrosine kinase activity, platelet-derived growth factor binding, and kinase inhibitor activity were dominant, indicating relevance to signaling pathways and therapeutic targets. The enrichment analysis underscores the involvement of key processes, components, and pathways in cancer progression, immune system regulation, and extracellular matrix interactions, offering potential insights into disease mechanisms and therapeutic targets. These processes are fundamental biological functions and pathways in BC and diabetes, as reported in several studies (48–54).

To identify DEGs between BC patients with and without diabetes, normalized expression data from the GEO database were analyzed separately for AA and EA cohorts. In the AA cohort, 3,245 DEGs were identified from 57 samples, including 1,922 upregulated and 1,323 downregulated genes, while in the EA cohort, 3,208 DEGs were detected across 17 samples, with 1,640 upregulated and 1,568 downregulated genes. A total of 786 DEGs were found to be common between the two cohorts. Key KEGG pathways identified in the AA cohort included cell cycle, ECM-receptor interaction, PI3K-Akt signaling, and AGE-RAGE signaling in diabetic complications. In contrast, significant pathways in the EA cohort included oxidative phosphorylation and diabetic cardiomyopathy. A Venn diagram illustrating the overlap between AA and EA transcriptomic profiles revealed 786 shared genes. Some of the key KEGG pathways, such as Notch signaling and Hippo signaling, both of which are relevant to breast cancer and diabetes (55–58).

The study analyzed WES data from 23 BC patients with diabetes, sourced from the NCBI SRA database, using a customized computational pipeline. Annotation via the Ensembl VEP database classified these variants into non-coding (56.9%) and coding (43.1%). Among coding variants, 96% were missense, 3% synonymous, and 1% stop-gained, with no start-lost variants detected. A total of 899 variants were analyzed, with no novel variants identified. After removing duplicates, these variants spanned 298 genes, which were reduced to 208 unique genes. A Venn diagram illustrated the overlap between transcriptomic and exomic datasets, identifying 2,804 genes unique to transcriptomics, 197 genes exclusive to exomics, and 11 common genes (Figure 6A). Two genes without mutations were excluded, leaving nine key genes: *SKI* (59, 60)*, TNFRSF1B* (61, 62)*, PDPN* (62, 63), *SLC25A34* (64), *EPHA2* (65, 66), *IFFO2* (67, 68), *ARHGEF16* (69, 70), *FBXO6* (71, 72), and *PADI2* (73, 74) for further analysis. Among these, six were upregulated, and three were downregulated. These genes play significant roles in both diabetes and BC. We analyzed gene expression across two cohorts—AA and EA populations and mapped these four genes (*SKI*, *TNFRSF1B*, *SLC25A34*, and *EPHA2*) that were consistently present in both groups.

Functional analysis of these nine genes revealed enriched terms across GO categories and Reactome pathways. GO-BP terms included processes like protein localization and synaptic pathways. GO-CC analysis highlighted synaptic and endosomal compartments, indicating roles in cellular organization and signaling. GO-MF terms included ubiquitin-protein ligase binding, TNF activity, chemokine activity, and cadherin binding, essential for protein regulation and interactions. These biological functions were enriched in BC and diabetes in other studies (75–79). Reactome pathways featured RHOG GTPase cycle, TNF-receptor binding, EPHA-mediated growth cone collapse, and other signaling pathways. Among these, the TNF pathway is significant in connecting BC and diabetes (16, 80, 81). The analysis focused on identifying the most deleterious variants using a comprehensive suite of online prediction tools. Among the nine genes analyzed, TNFRSF1B (L264P) and PDPN (A105G) were identified as the top two variants predicted to be most deleterious. These mutations remain poorly characterized and have not been extensively studied. TNFRSF1B (also known as TNFR2), a receptor for the pro-inflammatory cytokine TNF-*α*, is primarily expressed in immune cells, endothelial cells, and certain tumor cells, playing a pivotal role in immune regulation, inflammation, and cell survival. As chronic inflammation is a common feature of both BC and diabetes, TNFRSF1B may represent a molecular link between these diseases. It contributes to shared inflammatory pathways by promoting a pro-inflammatory microenvironment, and the presence of missense mutations in TNFRSF1B among BC patients with diabetes may exacerbate both tumor progression and metabolic dysfunction. Given its involvement in both cancer and metabolic disease, TNFRSF1B holds potential as a biomarker for identifying at-risk BC patients with diabetes and guiding personalized treatment strategies. Moreover, targeting TNFRSF1B signaling such as through TNF-*α* inhibitors could offer therapeutic benefits by mitigating inflammation and tumor development. Understanding genetic variations in TNFRSF1B may also inform precision medicine approaches that address the dual challenges of cancer and metabolic dysregulation (5, 16, 82–84).

**Figure 8:**
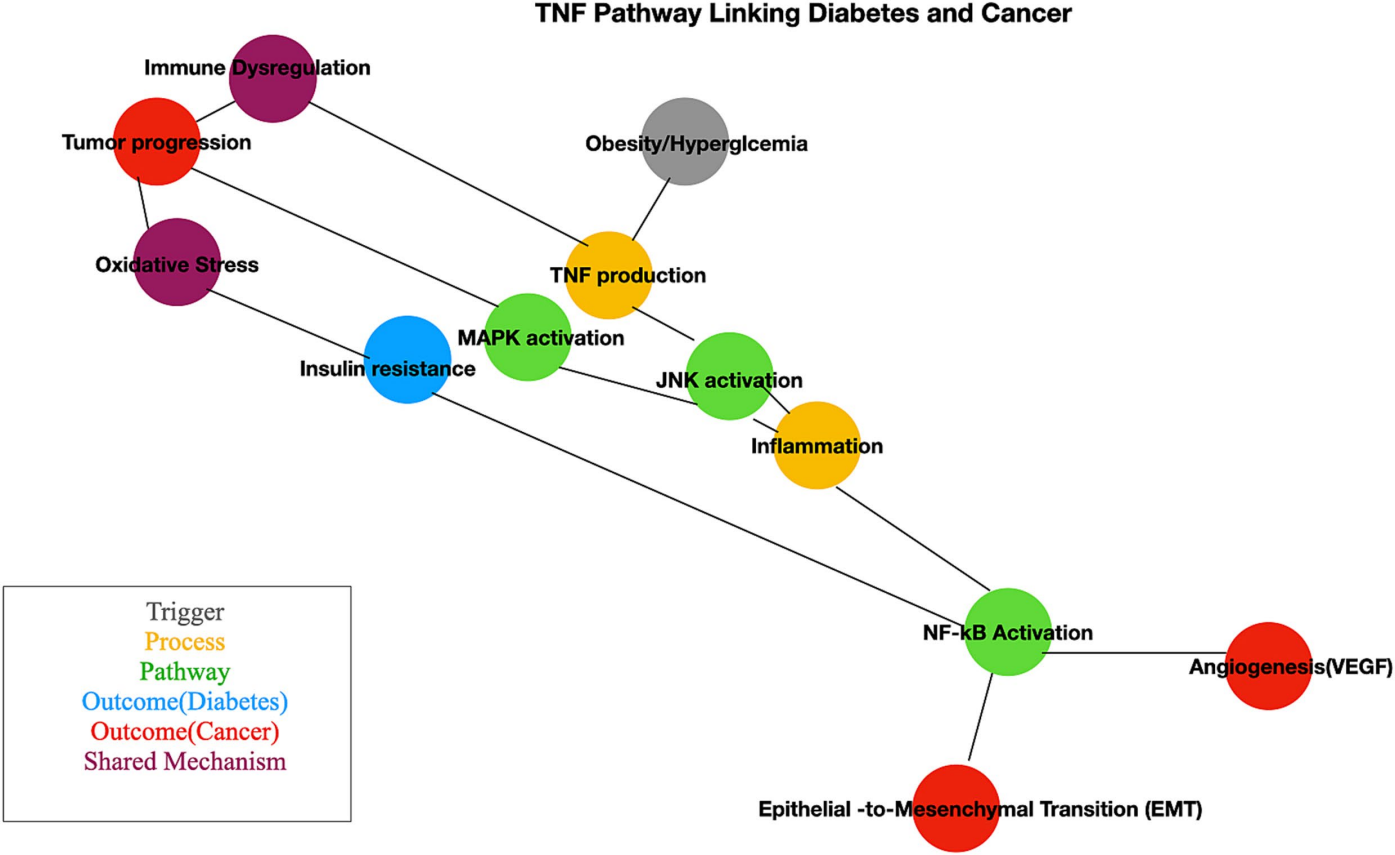
Pathway mechanism linked with diabetes and cancer. This schematic illustrates the interconnected molecular mechanisms linking diabetes and cancer. Key pathways include insulin signaling, chronic inflammation, oxidative stress, and altered metabolism. The figure highlights how hyperinsulinemia and insulin resistance influence cancer cell proliferation and survival through pathways like PI3K/AKT/mTOR and MAPK.

TNF pathway plays a crucial role in linking chronic inflammation, metabolic dysfunction, and cancer progression, providing an everyday mechanistic basis for its involvement in diabetes and BC. TNF, produced by adipocytes and macrophages in adipose tissue, is elevated in obesity and diabetes (85, 86). It inhibits insulin signaling by phosphorylating insulin receptor substrate-1 (*IRS1*), disrupting pathways essential for glucose uptake. TNF-induced NF-κB activation and oxidative stress exacerbate inflammation, worsening insulin resistance (87). TNF-mediated inflammation also contributes to beta-cell dysfunction, reducing insulin secretion. Prolonged TNF signaling increases circulating free fatty acids, further impairing metabolic homeostasis (88). In BC, chronic TNF secretion by cancer-associated macrophages and stromal cells creates a pro-inflammatory environment that supports tumor growth (89). NF-κB activation in cancer cells increases the expression of anti-apoptotic genes, helping tumor cells evade programmed cell death (90). TNF drives epithelial-to-mesenchymal transition (EMT), enhancing cancer cell motility and invasion, and promotes angiogenesis via VEGF induction, facilitating tumor vascularization and growth (91). The cross-talk between diabetes and BC with shared mechanisms. Obesity and hyperglycemia heighten TNF levels, creating a pro-inflammatory milieu (92). TNF exacerbates oxidative stress, which damages DNA and increases cancer risk (93). TNF-mediated immune suppression allows cancer cells to escape immune surveillance. Insulin resistance and hyperinsulinemia, driven by TNF, activate pathways like PI3K/AKT, promoting cancer cell proliferation (94). Elevated TNF levels in diabetic patients may accelerate BC progression through increased inflammation and angiogenesis (95, 96). These mechanisms are illustrated in a simplified manner in Figure 8.

Our analysis identifies the TNF pathway as a crucial mediator in the interplay between BC and diabetes. While pathways such as PI3K-AKT, JAK–STAT, and mTOR are also implicated, our differential expression analysis reveals a significant enrichment of TNF receptor activity among genes common to both conditions. This indicates that TNF signaling plays a pivotal role in inflammation, apoptosis, and immune regulation, potentially driving the interactions between these diseases. Although the PI3K-AKT and MAPK pathways contribute broadly, TNF signaling stands out as a central hub, highlighting its potential as a therapeutic target (5, 50, 97). Further studies are needed to refine these insights. Targeting the TNF gene or its variants could have substantial therapeutic implications, especially for research on comorbidities. Anti-TNF therapies could reduce inflammation, benefiting patients with both metabolic disorders and cancer. Combining TNF inhibitors with treatments specific to metabolic or cancer conditions may offer synergistic benefits, particularly for patients with both diabetes and BC. The TNF pathway exemplifies how chronic inflammation is a common factor in complex diseases like diabetes and BC, emphasizing the importance of addressing systemic inflammation in therapeutic strategies.

## 5 Conclusion

This study provides a comprehensive examination of the biomarker landscape in BC associated with diabetes through integrative transcriptomics and exome analysis. Utilizing computational approaches, we identified key differentially expressed genes, mutations, and genes with potential deleterious variants that may elucidate the interplay between these conditions. Our findings highlight potential biomarkers and therapeutic targets that could enhance stratification, diagnosis, and treatment for patients with comorbid BC and diabetes. Future studies validating these biomarkers in experimental and clinical settings could significantly advance our understanding and management of this complex disease intersection.

### 5.1 Limitation of the study

We acknowledge the limitation of our Whole Exome Sequencing (WES) analysis due to the relatively small sample size (*n* = 23). This constraint primarily arises from our focus on integrating transcriptomic and exomic data specifically for BC patients with diabetes, ensuring a well-defined cohort for robust multi-omics analysis. Additionally, the stringent patient selection criteria and data availability restricted our analysis to tumor samples alone, as paired normal controls were not available within the dataset.

## Data Availability

The datasets presented in this study can be found in online repositories. The names of the repository/repositories and accession number(s) can be found in the article/Supplementary material.

## Supplementary material

SUPPLEMENTARY FIGURE 1 **(A)** Selection of samples from transcriptomics data. **(B)** Selection of samples from exomic data.

SUPPLEMENTARY FIGURE 2 Statistical plots of transcriptomics data. The boxplot represents the distribution of normalized transcriptomics data across all samples. Each box corresponds to an individual sample, with the central line representing the median expression level. **(A)** Boxplot of AA cohort; **(B)** Boxplot of EA cohort. The UMAP plot illustrates the clustering of transcriptomics data, with each point representing an individual sample. Samples are color-coded based on their respective groups (BC with diabetes vs BC without diabetes). **(C)** UMAP of AA cohort; **(D)** UMAP of EA cohort.

SUPPLEMENTARY FIGURE 3 The figure presents the functional enrichment analysis of DEGs identified from transcriptomic data of the AA cohort. Panels include **(A)** GO-BP, **(B)** GO-CC, **(C)** GO-MF, and **(D)** KEGG pathways. Bar lengths represent both statistical significance (adjusted *p*-values) and gene ratios, offering insights into the molecular roles and functional relevance of the DEGs.

SUPPLEMENTARY FIGURE 4 The figure presents the functional enrichment analysis of DEGs identified from transcriptomic data of the AA cohort. Panels include **(A)** GO-BP, **(B)** GO-CC, **(C)** GO-MF, and **(D)** KEGG pathways. Bar lengths represent both statistical significance (adjusted *p*-values) and gene ratios, offering insights into the molecular roles and functional relevance of the DEGs.

SUPPLEMENTARY FIGURE 5 This figure displays the overlapping genes identified through a transcriptomics analysis of the AA and EA cohort. These shared genes represent a subset with potential biological significance and functional relevance. **(A)** 786 overlapping genes were present in both cohorts. The functional analysis of common genes was performed. **(B)** GO-BP, **(C)** GO-CC, and **(D)** GO-MF. **(E)** KEGG pathways.

SUPPLEMENTARY TABLE 1 Metadata information of transcriptomics data.

SUPPLEMENTARY TABLE 2 Detailed metadata information of AA and EA cohorts.

SUPPLEMENTARY TABLE 3 Detailed information of differentially expressed genes (pooled cohort) in comparison of BC with and without diabetes.

SUPPLEMENTARY TABLE 4 Detailed information of differentially expressed genes of the AA cohort in comparison of BC with and without diabetes.

SUPPLEMENTARY TABLE 5 Detailed information of differentially expressed genes of the EA cohort in comparison of BC with and without diabetes.

SUPPLEMENTARY TABLE 6 Detailed information of missense data.
