# Supplementary figures and images for: Multi-omics insights into biomarkers of breast cancer associated diabetes: a computational approach
Source: Front Med (Lausanne). 2025 Jun 6;12:1572500. doi: 10.3389/fmed.2025.1572500 (PMC12179083; doi:10.3389/fmed.2025.1572500)

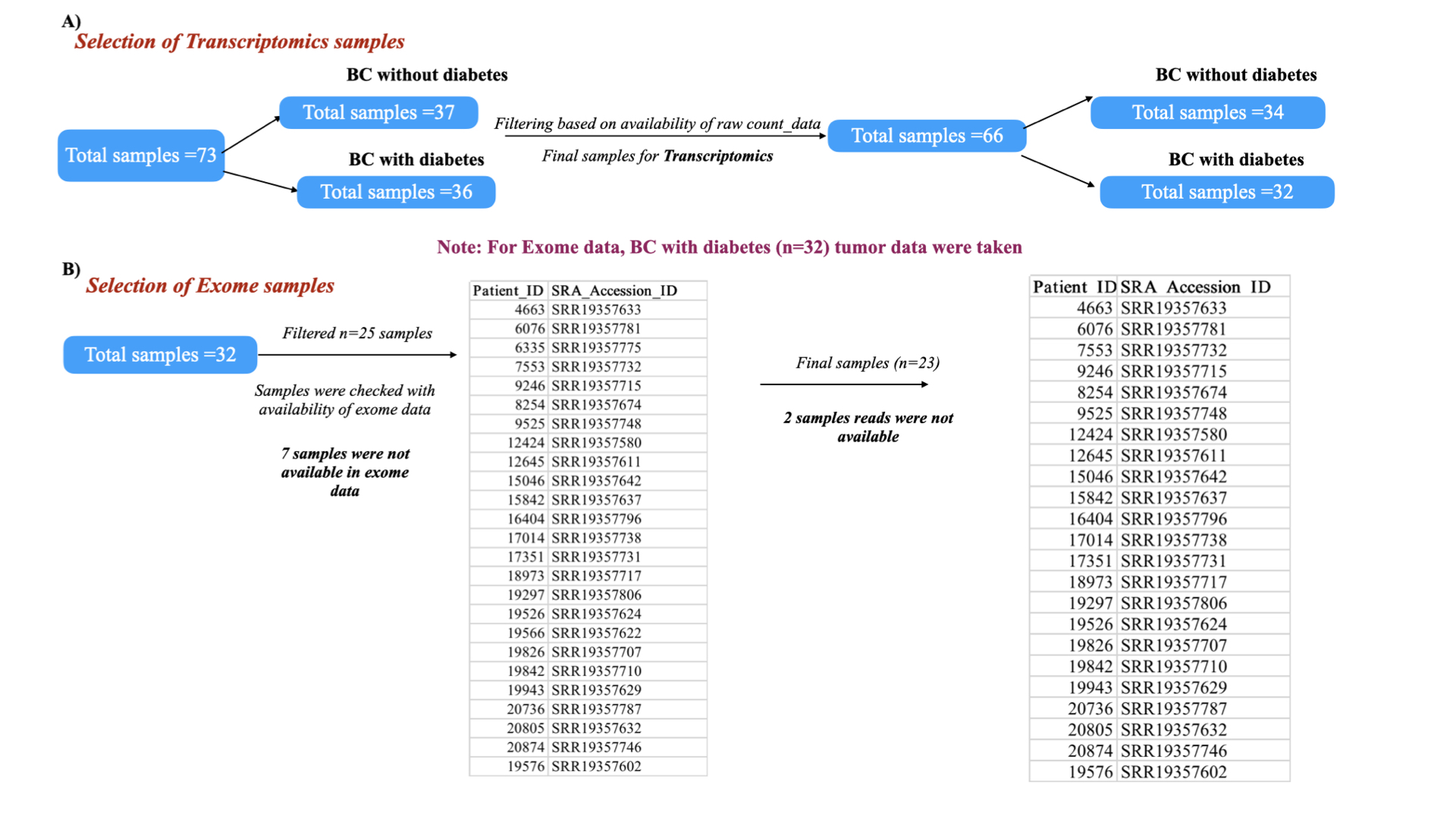

Supplement: SUPPLEMENTARY FIGURE 1 — (A) Selection of samples from transcriptomics data. (B) Selection of samples from exomic data. [file Image_1.jpeg]

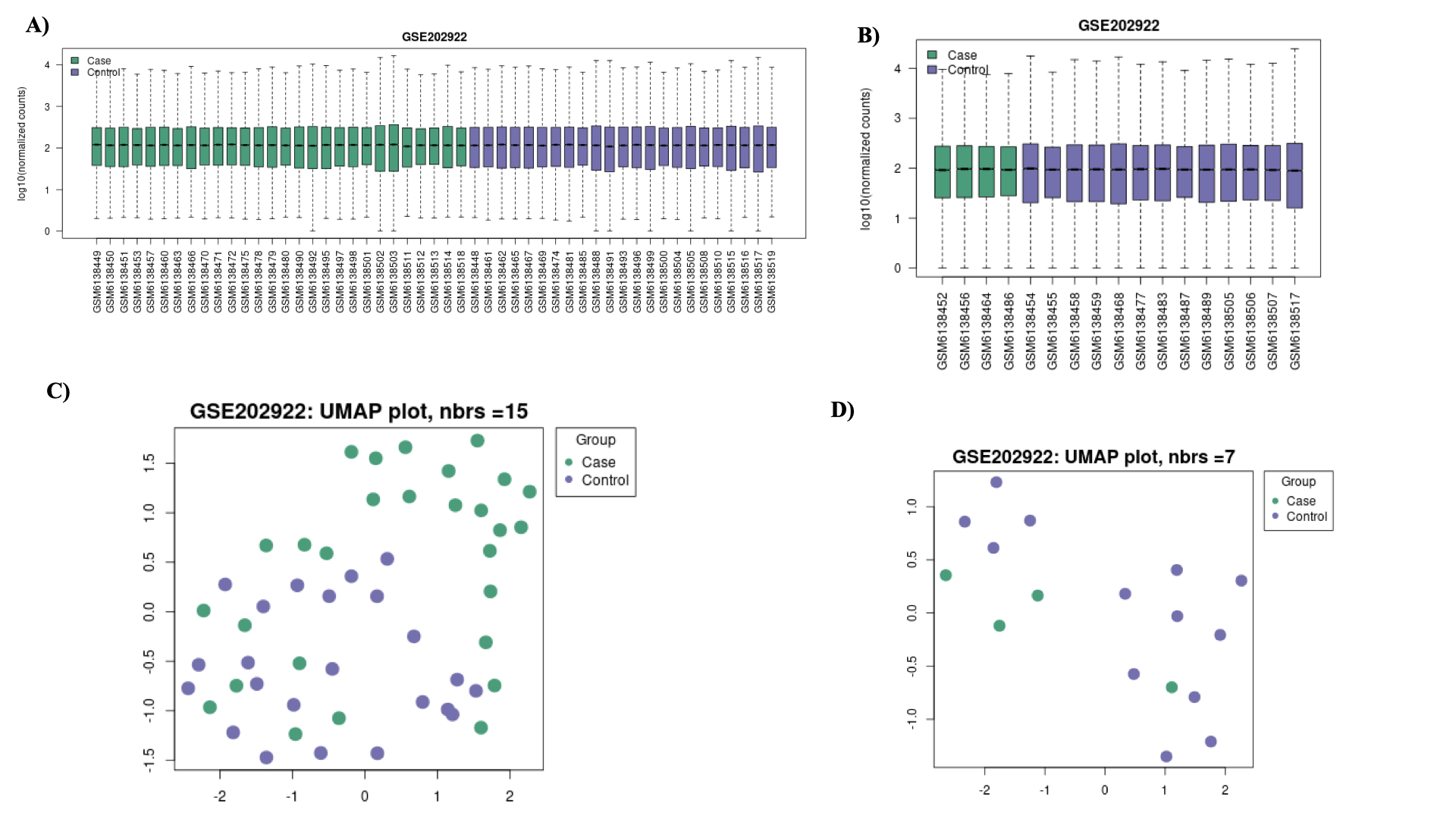

Supplement: SUPPLEMENTARY FIGURE 2 — Statistical plots of transcriptomics data. The boxplot represents the distribution of normalized transcriptomics data across all samples. Each box corresponds to an individual sample, with the central line representing the median expression level. (A) Boxplot of AA cohort; (B) Boxplot of EA cohort. The UMAP plot illustrates the clustering of transcriptomics data, with each point representing an individual sample. Samples are color-coded based on their respective groups (BC with diabetes vs BC without diabetes). (C) UMAP of AA cohort; (D) UMAP of EA cohort. [file Image_2.jpeg]

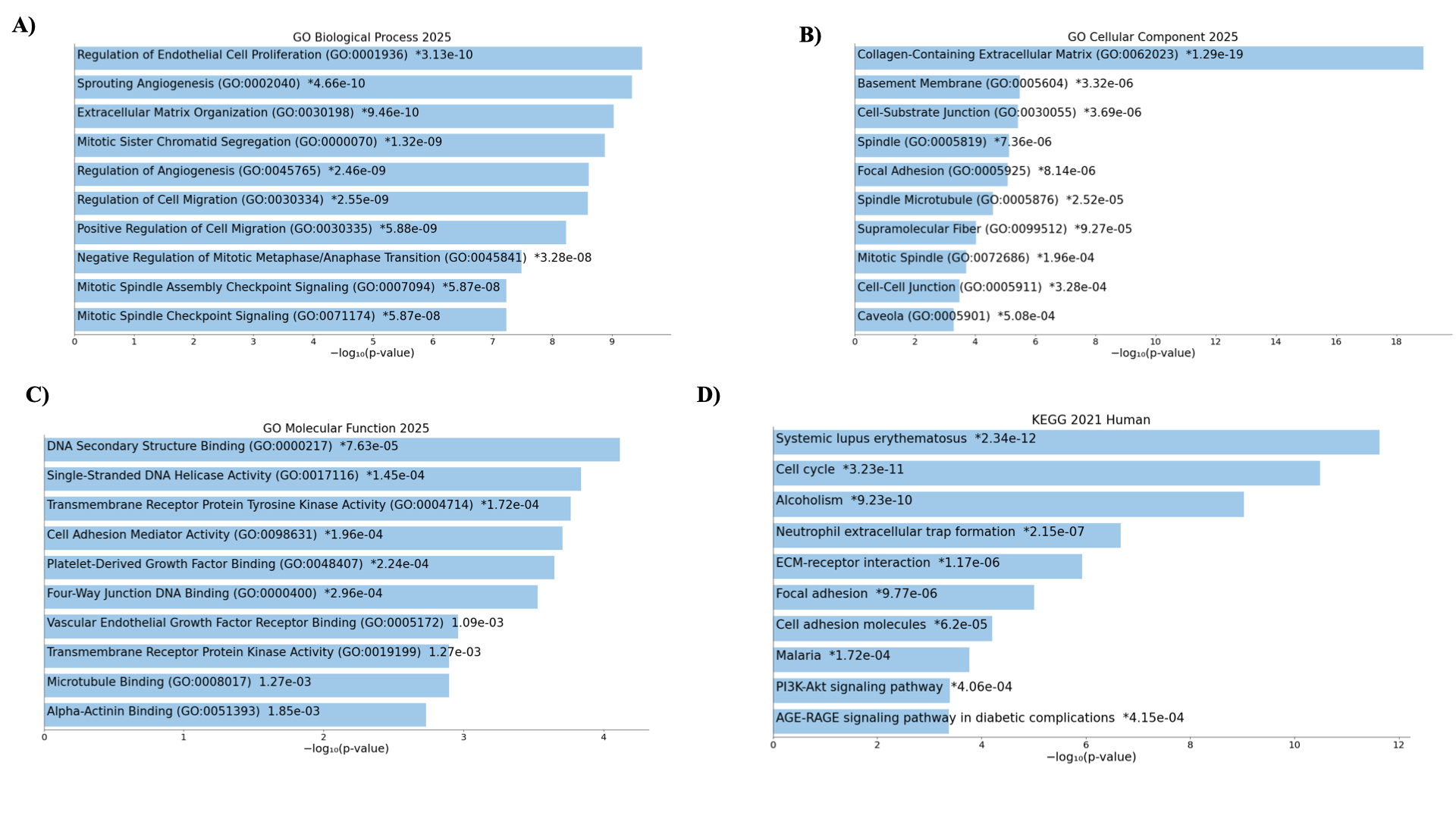

Supplement: SUPPLEMENTARY FIGURE 3 — The figure presents the functional enrichment analysis of DEGs identified from transcriptomic data of the AA cohort. Panels include (A) GO-BP, (B) GO-CC, (C) GO-MF, and (D) KEGG pathways. Bar lengths represent both statistical significance (adjusted p-values) and gene ratios, offering insights into the molecular roles and functional relevance of the DEGs. [file Image_3.jpeg]

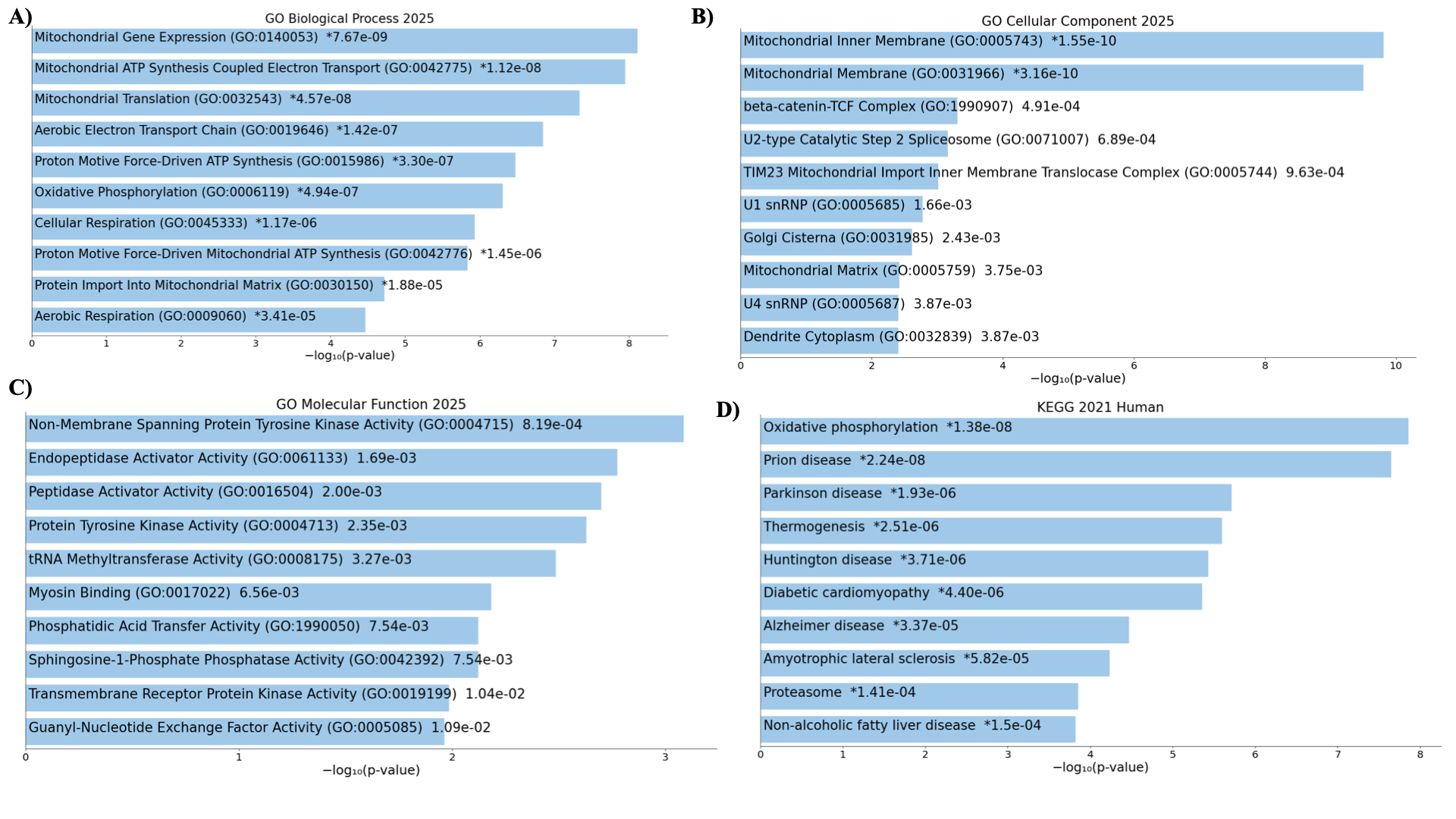

Supplement: SUPPLEMENTARY FIGURE 4 — The figure presents the functional enrichment analysis of DEGs identified from transcriptomic data of the AA cohort. Panels include (A) GO-BP, (B) GO-CC, (C) GO-MF, and (D) KEGG pathways. Bar lengths represent both statistical significance (adjusted p-values) and gene ratios, offering insights into the molecular roles and functional relevance of the DEGs. [file Image_4.jpeg]

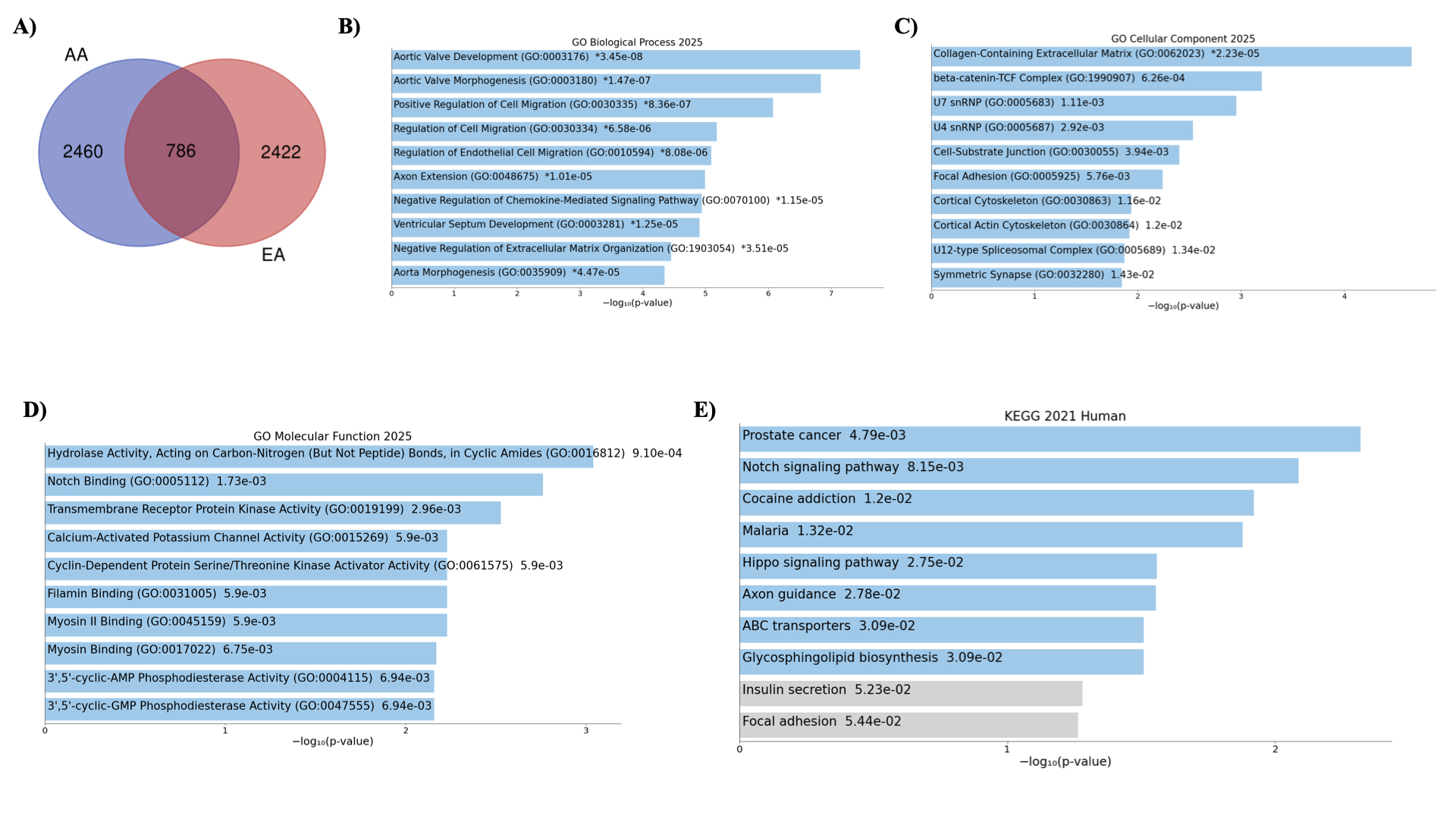

Supplement: SUPPLEMENTARY FIGURE 5 — This figure displays the overlapping genes identified through a transcriptomics analysis of the AA and EA cohort. These shared genes represent a subset with potential biological significance and functional relevance. (A) 786 overlapping genes were present in both cohorts. The functional analysis of common genes was performed. (B) GO-BP, (C) GO-CC, and (D) GO-MF. (E) KEGG pathways. [file Image_5.jpeg]
